# Photoluminescence of PdS_2_ and PdSe_2_ quantum dots†

**DOI:** 10.1039/c9ra07445g

**Published:** 2019-11-21

**Authors:** Xinyu Wang, Wayesh Qarony, Ping Kwong Cheng, Mohammad Ismail, Yuen Hong Tsang

**Affiliations:** The Hong Kong Polytechnic University Shenzhen Research Institute Shenzhen Guangdong China yuen.tsang@polyu.edu.hk; Department of Applied Physics and Materials Research Centre, The Hong Kong Polytechnic University Hung Hom Kowloon Hong Kong China

## Abstract

Group-10 transition metal dichalcogenide (TMD) materials have recently attracted considerable attention in optoelectronics applications. However, so far their quantum dot (QD) counterparts with photoluminescence (PL) nature still remain to be revealed. In this study, 2 typical types of group-10 TMD material (PdS_2_ and PdSe_2_) QDs are fabricated *via* liquid exfoliation using *N*-methyl-2-pyrrolidone (NMP) solvent. The absorption and PL spectra of these QD solutions are studied, exhibiting excitation wavelength-dependent behaviors and large Stokes shifts. Furthermore, the quantum yield and decay lifetime are also investigated and analyzed. The obtained results suggest promising optoelectronic applications with group-10 TMD QDs in the future.

## Abstract 2

PdS_2_ and PdSe_2_ QDs are fabricated *via* liquid exfoliation using NMP solvent. The PL behaviors of these QD solutions are studied. The obtained results suggest promising optoelectronic applications with group-10 TMD QDs in the future.

## Graphical abstract

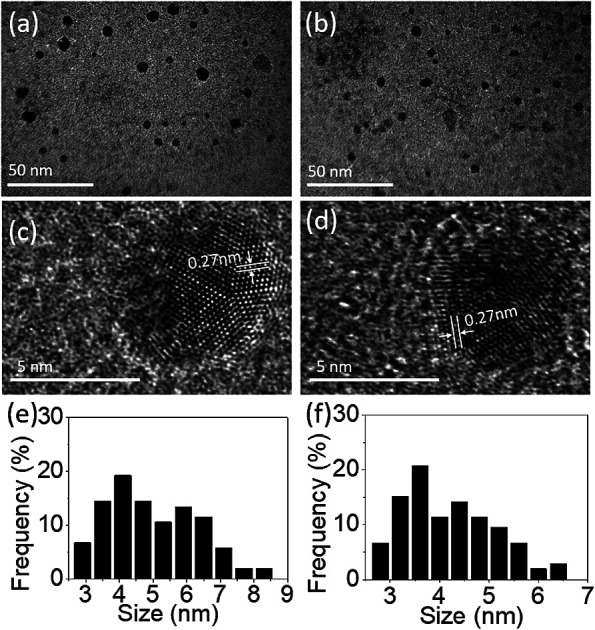

## 1. Introduction

The lack of an intrinsic band gap in graphene has stimulated research into alternative two-dimensional (2D) materials for semiconductor applications.^1^ Recently, traditional transition-metal dichalcogenide (TMD) semiconductors have become very promising candidates among the 2D materials series owing to the direct band gap behavior in monolayer TMDs at the *K* point,^2^ along with excellent electronic, catalytic, optical, and mechanical properties.^3–12^ Furthermore, TMD materials exhibit superior photoluminescence behavior,^3,4^ piezoelectric properties,^13,14^ and controllable optical performance by modulating valleys in the *k* space.^15–18^ The development of several optoelectronic devices, such as strain sensors,^19^ transistors,^20^ and highly sensitive and broadband photodetectors^21–23^ will highly benefit from the assistance of these inherent properties.

The research frontiers in TMDs are mainly focused on investigating novel semiconductor characteristics by combining various transition metal atoms with chalcogens (S, Se, and Te).^2^ Several noble metal atoms, such as platinum (Pt) and palladium (Pd) can bond to these four chalcogens, resulting in layer structure. In the material's atomic structure of such TMDs, each unit cell consists of a noble metal atom sandwiched by 2 chalcogen atoms, leading to the formation of a hexagonally plain structure.^24–29^ Unlike to the traditional TMDs materials these advanced TMDs materials again exhibit several unique material properties, such as superconductivity, high carrier mobility, broad tunable band gap, and stability in the air.^26,28–31^ The application of such materials in optoelectronic devices is also wide with remarkable performances. For instance, PtS_2_ with (0.25 (bulk) to 1.6 eV (monolayer)) indirect band gap^28,29^ has been applied in field-effect transistor (FET),^29^ Q-switched laser,^25^ and catalysis.^32^ PtSe_2_, with a tunable indirect band gap from 0 (bulk) to 1.2 eV (monolayer), has also been used in saturable absorber^33^ and broadband photodetectors.^34,35^ Then electronic, optical, and catalytic properties with bandgap tunability have also been studied in PdS_2_ under tensile strain, exhibiting semi-metallic characteristic even for the bilayer structure.^36^ Furthermore, the indirect bandgap with 1.0 eV is found to appear in PdS_2_ when it reaches monolayer thickness, whereas the metallic characteristics are exhibited for its bulk counterpart.^36^ A PdS_2_ logical junction has also recently been reported with 2.5 nm channel length and a gate voltage dependent *I*–*V* characteristic.^37^ Additionally, indirect bandgap PdSe_2_ with a bandgap of 1.31 eV has been exhibited for its monolayer structure and metallic for the bulk counterpart.^26^ The applications of PdSe_2_ based thermal electricity,^38^ FET,^39^ and photodetector^40^ suggest its exceptional optoelectronics properties. In the beginning, the PtS_2_ was anticipated and experimentally demonstrated as that the photoluminescence (PL) signal is “too weak to be detected” owing to its indirect bandgap, even for the case of its monolayer structure.^28^ Nonetheless, the PL behavior of PtS_2_ has successfully observed *via* scaling down to quantum dots (QDs).^41^ However, the PL behaviors of PdS_2_ and PdSe_2_ are still remained obscure. Since PdS_2_ and PdSe_2_ exhibit similar crystal structure with PtS_2_, including the indirect bandgap, such group-10 TMDs materials arouse our interest for the investigation of PL performance.

QDs are defined when materials reach a size smaller than twice of their excitonic Bohr radius. QDs have been reported as a novel structure with exceptional large transition energy in comparison to their bulk or layer counterpart, resulting in enhanced PL performance of the materials; thanks to quantum confinement effect.^42–44^ In a previous study, we successfully fabricated PtS_2_ QDs *via* low cost liquid exfoliation with water.^41^ Herein, 2 typical group-10 TMDs materials, PdS_2_ and PdSe_2_, QDs are fabricated with the aid of similar liquid exfoliation technique, while *N*-methyl-2-pyrrolidone (NMP) is selected as solvent for its better compatibility of surface energy as compared to the water. PtS_2_ QDs solution is also fabricated with same method in order to assist analyzation by comparing with PdS_2_ and PdSe_2_ QDs. To our knowledge, this is the first experimental demonstration of optical characteristics of PdS_2_ and PdSe_2_ QDs, where the UV-Vis spectra, PL spectra and the decay lifetimes of PtS_2_ QDs, PdS_2_ QDs, and PdSe_2_ QDs are monitored and analyzed by comparing with each other.

## 2. Experimental section

### 2.1 Materials fabrication

In this study, PdS_2_, PdSe_2_, and PtS_2_ QDs were fabricated *via* liquid exfoliation from their bulk powder raw materials. NMP was selected to be the solvent for its compatible surface energy, resulting in stabilizing and preventing TMDs nanoparticles from agglomeration. In the first step of the fabrication process, a 50 mg of PtS_2_ raw material (Alfa Aescar) was poured into 250 mL of NMP solution. Next, a probe sonication was applied to the mixture under 250 W power with 20 kHz frequency for about 3 hours long, while 27 °C temperature was maintained throughout the process. In this case, the operation time of ultrasonic probe was set to 2 s at an interval of 4 s. Then, 2/3 of dispersion close to the liquid level was taken and stirred for 6 h under 140 °C. The supernatant liquor was obtained by centrifugation at 2000 rpm for 5 min to separate QDs from bulky raw materials. This QDs as-prepared solution was taken for the experiments and characterizations.

### 2.2 Characterization

The high-resolution images and lattice fringes of as-prepared QDs were observed *via* Transmission Electron Microscopy (TEM, Jeol JEM-2100F). The thickness of QDs was scanned under tapping mode with the aid of Atomic Force Microscopy (AFM, Bruker Nanoscope 8). Each of the AFM samples for QDs were prepared *via* drop casting the as-prepared QDs solution on the surface of quartz substrate followed by drying under 80 °C in the air atmosphere. Element contained in as-prepared solution is confirmed by the Energy Dispersive X-ray Spectroscopy (EDX, ULTIM MAX 170) performed on Scanning Electron Microscopy (SEM, GeminiSEM 300). The samples for EDX and SEM measurements are fabricated by drop-casting on the silicon substrate following by drying under the air atmosphere. Chemical composition of raw materials was detected by X-ray photoelectron spectroscopy (XPS, ESCALAB 250Xi, Thermo Fisher Scientific) with achromatic 200 W Al Kα as the X-ray source and the resolution of 0.10 eV. Before conducting the XPS measurement, the powders of raw materials were utilized to eliminate water in the oven under 60 °C for 3 h. The PL measurement was performed with Edinburgh CD920. The emission spectra of QDs solution were monitored on excitation at given wavelength under ambient environment. The excitation source was Xenon lamp. Time-resolved measurements were monitored by HORIBA FluoroMax-4 spectrometer for ultrafast studies and time-correlated single-photon counting (TCSPC) accessory for lifetime determinations. UV-Vis absorption spectroscopy was recorded by Shimadzu UV-2550.

## 3. Results and discussion

**Fig. 1:**
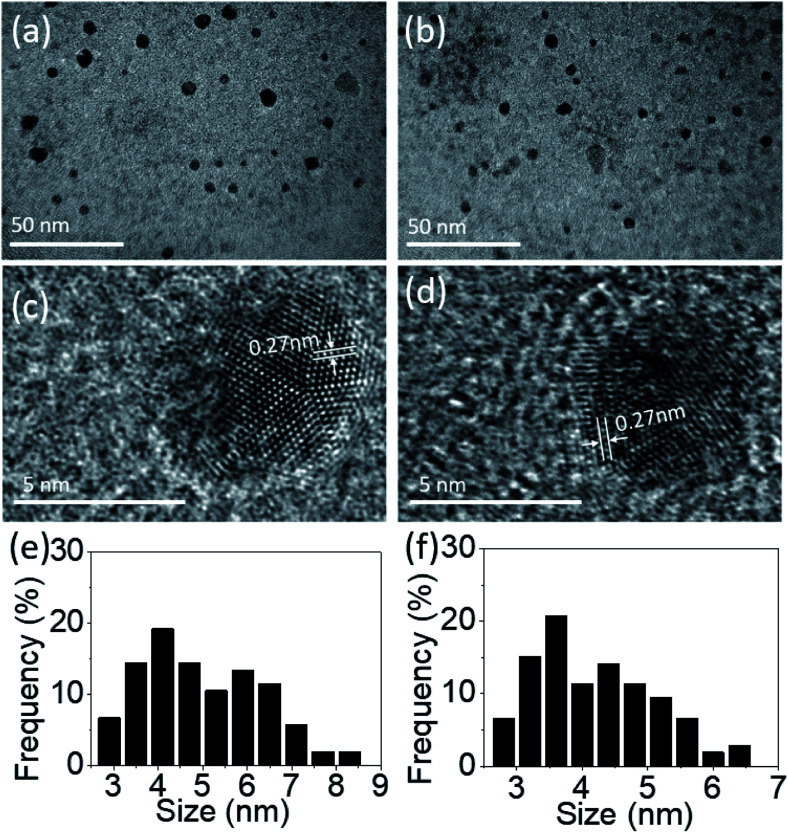
TEM images of (a) PdS_2_ QDs solution and (b) PdSe_2_ QDs solution; high resolution TEM images of a typical (c) PdS_2_ QD and (d) PdSe_2_ QD; and size distributions based on multiple images of (e) PdS_2_ QDs and (f) PdSe_2_ QDs.

Fig. 1 is an illustration of TEM images for PdS_2_, and PdSe_2_ group-10 TMDs materials QDs. The TEM samples were prepared by drop-casting as-prepared QDs suspensions on holey copper grid, followed by a drying process in the ambient environment under 60 °C for 1 h. The morphologies and scattered distributions of PdS_2_, PdSe_2_, and PtS_2_ QDs can be clearly observed from Fig. 1e and f, and S1c,† respectively. The statics of the corresponding size distributions are depicted in Fig. 1c and d, and S1b† for number of 104 PdS_2_, 106 PdSe_2_ QDs, and 143 PtS_2_, respectively, pronouncing a statistical estimation of their average diameters of 4.99, 4.17, and 4.73 nm. High resolution images of PdS_2_, PdSe_2_, and PtS_2_ QDs were captured in Fig. 1c and d, and S1b,† respectively, indicating the high crystallinity of the as-prepared samples. For the PdS_2_ QDs with a determined lattice distance of 0.27 nm is corresponding to the (200) plane, which agrees well with a simulated data as recently reported in the literature.^36^ Then the PdSe_2_ QDs shows a 0.27 nm lattice periodicity that matches its (200) crystalline plane.^24^ The interplanar spacing of 0.29 nm for PtS_2_ QDs as observed in Fig. S1b† is corresponding to (100) plane.^28,29,41^ The results imply that the ultrasonic energy is sufficient to separate layered group-10 TMDs materials from the van der Waals forces and even splits them into nanoscales. Thanks to the outstanding stability of group-10 TMDs materials, which allows to obtain good crystallinity in the materials.

**Fig. 2:**
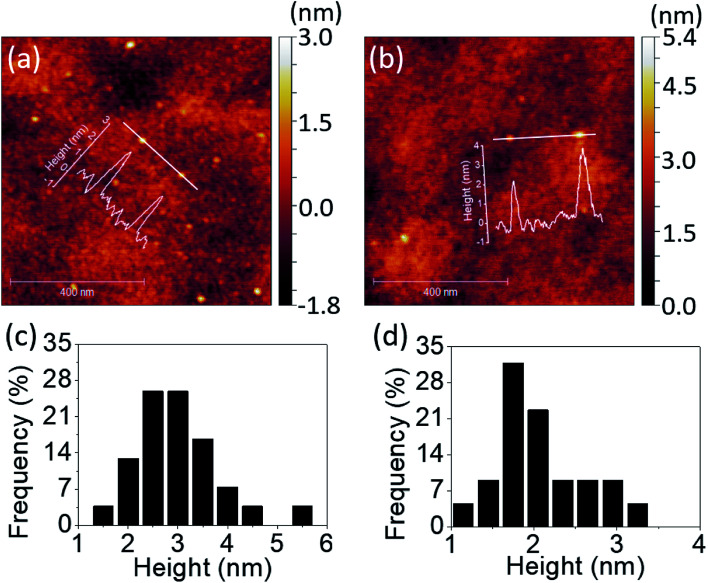
Line profiles and corresponding AFM images of (a) PdS_2_ QDs, and (b) PdSe_2_ QDs; height distributions based on several images of (c) PdS_2_ QDs, and (d) PdSe_2_ QDs.

Further investigation on QDs' height profiles and height distributions were conducted *via* AFM measurement as shown in Fig. 2. The QDs were scattered on the quartz substrates by scanning processes. The scan size was set to be 1 × 1 μm^2^ and the observation of QDs was taken place randomly on the substrates. The height variation of PdS_2_, PdSe_2_, and PtS_2_ QDs can be clearly distinguished along line profiles as depicted in Fig. 2a and b, S2a,† respectively. The statics of 54 PdS_2_ QDs are shown in Fig. 2c, indicating an average height of 2.61 nm, which can be considered as 6 to 7 layers.^27^ The height information of 25 observed PdSe_2_ QDs is recorded in Fig. 2d. The thickness of such QDs are assumed to be 4 to 5 layers referring to 1.99 nm average height.^27^ The average height of 38 PtS_2_ QDs is determined to be 2.82 nm, suggesting 5 to 6 layers' thickness,^28^ and their height distribution is depicted in Fig. S2b.†

**Fig. 3:**
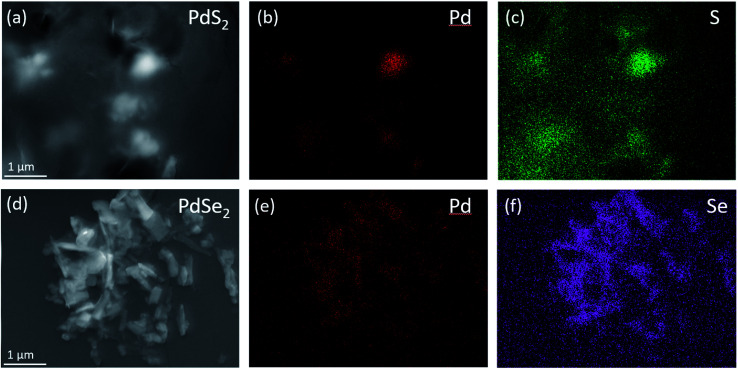
(a) SEM image of PdS_2_ QDs solution drop-casting on silicon substrate; (b and c) EDX elemental maps of Pd and S elements in (a); (d) SEM image of PdSe_2_ QDs solution drop-casting on silicon substrate; (e and f) EDX elemental maps of Pd and Se elements in (d).

The element compositions of as-prepared QDs solutions are detected *via* the Scanning Electron Microscopy (SEM) and the Energy Dispersive X-ray Spectroscopy (EDX) mapping. The mapping analysis shown in Fig. 3a–c confirms the existence of Pd and S elements in PdS_2_ QDs solution. The consistent distributions of Pd and Se shown in Fig. 3d–f verify the elements in PdSe_2_ QDs solution. The element mapping images in Fig. S3† evident the elements contained in PtS_2_ QDs solution since both Pt and S are accordantly distributed along the film surface.

**Fig. 4:**
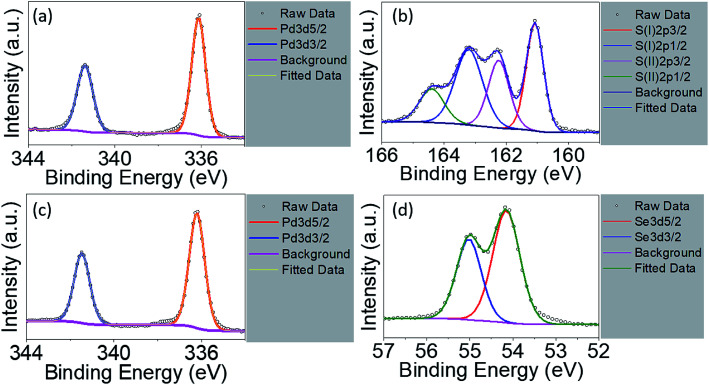
High resolution X-ray photoelectron spectra of PdS_2_ for (a) Pd3d region and (b) S2p region; PdSe_2_ for (c) Pd3d region and (d) Se3d region.

The EDX is not suitable used as quantitative analysis method. Therefore, the XPS measurement was conducted on the raw powder materials counterparts in order to investigate their chemical compositions as shown in Fig. 4. For Pt4f spectra of PtS_2_, deconvolution of the line shape reveals two doublets, Pt^2+^ and Pt^4+^, essential to reconstruct Pt signals, as shown in Fig. S4a.† Pt^2+^ with level positions of Pt4f_7/2_ and Pt4f_5/2_ are fitted at 71.98 eV and 75.38 eV, along with Pt^4+^4f_7/2_ and Pt^4+^4f_5/2_ fitted at 71.98 eV and 75.38 eV, respectively. S2p deconvolution as shown in Fig. S4b† can be conducted showing one doublets: S2p_3/2_ at 162.78 eV and S2p_1/2_ at 164.18 eV. The signal with 168.08 eV binding energy refers to sulphate series constituting HSO_4_^−^ or SO_4_^2−^.^32,45^ Such considerable intensity of sulphate signal indicates partial oxidation on the surface of the PtS_2_ raw materials, resulting in Pt^2+^ as predominant doublet. The atomic percentage of Pt and S is determined to be 17.79% and 27.97%. The stoichiometric ratio is obtained as less than 0.5, which may be due to the origination of sulphate series from surface oxidation during drying process. For PdS_2_, signals of Pd3d_5/2_ and Pd3d_3/2_ are shown in Fig. 4a with binding energies of 336.08 eV and 341.38 eV. The S2p spectra for PdS_2_ can be divided into two chemical states^46^ as shown in Fig. 4b. For the case of PtS_2_ raw materials, S(i)2p_3/2_ and S(i)2p_1/2_ are simulated at 161.08 eV and 163.38 eV, while S(ii)2p_3/2_ and S(ii)2p_1/2_ with 162.28 eV and 164.38 eV exhibit similar to the S2p spectra. PdS_2_ stored under ambient conditions with no detectable sulphate series signal implies its high stability. The atomic percentage of Pd and S are found to be 3.08% and 6.96% on the surface, exhibiting a stoichiometric ratio very close to 1/2 due to probably the absence of oxidation. The level positions of Pd3d_5/2_ and Pd3d_3/2_ in PdSe_2_ with 336.18 eV and 341.48 eV as depicted in Fig. 4c agree well with the presence of Pd signals in PdS_2_. The deconvolution of Se element gives rising to Se3d_5/2_ signal at 54.18 eV and Se3d_3/2_ signal at 54.98 eV as shown in Fig. 4d. The atomic percentage of 6.76% and 15.11% for the Pd and Se elements support the idea of air stability as recently reported in the literature.^26^

**Fig. 5:**
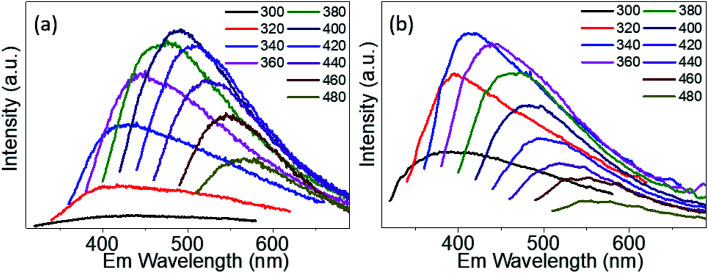
Emission (Em) spectra of (a) PdS_2_ QDs solution, and (b) PdSe_2_ QDs solution.

The PL emission spectra of PdS_2_, and PdSe_2_ QDs show excitation wavelength-dependent behaviors, *i.e.* the wavelength of PL emission peak red-shifts under crescent excitation wavelength, which have been widely reported by several studies on semiconductor QDs,^47,48^ traditional TMDs QDs,^42,44^ and carbon QDs.^49,50^ Due to quantum size effect, the poly-diversity of QDs sizes give rising to the variation of band gap energies. The larger QDs exhibit lower band gap energies, and *vice versa*. The PL spectra of PdS_2_ QDs as shown in Fig. 5a were observed from 400 nm to 567 nm under the excitation ranging from 320 nm to 480 nm. The highest PL peak is determined to be 491 nm for the excitation wavelength of 400 nm. The PL spectra of PdSe_2_ are depicted in Fig. 5b, displaying emission peaks ranging 379 nm to 551 nm, while the excitation wavelength was being tuned from 300 nm to 480 nm. The 340 nm excitation wavelength presents a maximum emission peak at 413 nm. The PtS_2_ QDs sample exhibits PL peak wavelength ranging from 381.5 to 548 nm while excitation wavelength shifts from 300 nm to 480 nm as shown in Fig. S5.† The maximum emission peak could be found at 468.5 nm for the excitation wavelength of 380 nm. The maximum emission peak wavelengths demonstrate a trend of PdSe_2_ QDs < PdS_2_ QDs, which might be owing to including but not limited to their intrinsic chemical property. The intrinsic bandgaps may also play important role in defining the emission peak wavelength. Since monolayer PdS_2_ holds a smaller bandgap than monolayer PdSe_2_, the PdS_2_ sample exhibits a substantial redshift *vs.* PdSe_2_ sample. This PL emission peak trend also accords with their average sizes determined *via* TEM characterizations, which may further prove the quantum size effect.

**Fig. 6:**
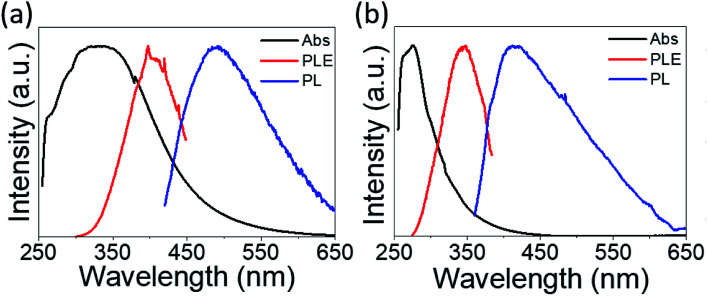
Normalized absorption spectra (black curve) for (a) PdS_2_ QDs and (b) PdSe_2_ QDs solutions. Photoluminescence excitation (PLE) spectra (red curve) for (a) 491 and (b) 413 nm emission wavelengths, which are observed at the peaks of the corresponding emission spectra (blue curve).

The absorption spectra are depicted in Fig. 6 with black lines. The distinct absorption peaks at 330, 274, and 307 nm can be observed in Fig. 6a and 5b, and S5† for the solutions of PdS_2_, and PdSe_2_, and PtS_2_ QDs, respectively. The maximum emission peaks (in blue lines) for all QDs samples and their corresponding photoluminescence excitation (PLE) spectra (in red line) are also displayed in Fig. 6. The maximum PLE peaks for PdS_2_, and PdSe_2_, and PtS_2_ QDs samples exhibit at 397.5, 346, and 377 nm as shown in Fig. 6a and b, and S6,† respectively. These PLE peaks agree well with the previous PL emission analysis, implying to 0.59 eV, 0.58 eV, and 0.64 eV stokes shifts for the PdS_2_, PdSe_2_, and PtS_2_, respectively. Such large stokes shifts are caused by the quantum confinement effect when materials size approaches to the atomic scale, resulting in dramatic expansion of spatial overlaps for electron–hole wave functions.^51^ The splitting of the lowest singlet fine structure states and highest triplet states grows much larger comparing to their layers or bulky structure.^51,52^ The dark exciton ensues when excitation experiences a rapid thermalization process from active singlet state to passive triplet state, leading to red shift of PL emission spectrum and larger stokes shift. Quantum yield (QY) of PdS_2_, PdSe_2_, and PtS_2_ QDs are evaluated to be 11.11%, 7.18%, and 14.42%, respectively and tabulated in Table 1. The PL QY for as-prepared QDs samples are calculated by eqn (1).1
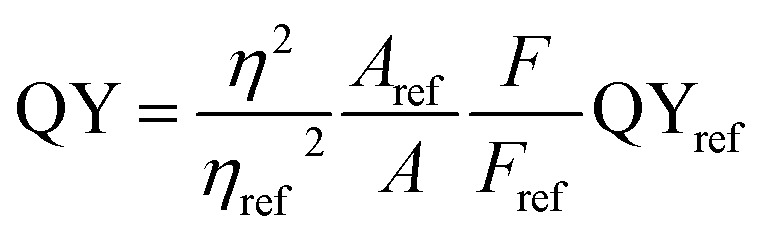
where, *η* represents the refractive index of the solvent. The quinine sulfate is dissolved in H_2_SO_4_ to prepare reference solution by controlling its absorbance similar to the as-prepared PdS_2_, PdSe_2_, and PtS_2_ solutions. Quinine sulfate solution holds refractive index of 1.33, while the QDs is dissolved in NMP solution which holds a refractive index of 1.47. *F* and *A* are referring to the integrated emission intensity and absorbance, respectively for samples and reference compound.

**Table 1:** Quantum yields of PtS_2_ QDs, PdS_2_ QDs, and PdSe_2_ QDs solutions

	*A*	*F*	*η*	QY
Quinine sulfate	0.024	6 095 040	1.33	54%
PtS_2_ QDs	0.036	1 459 610	1.47	14.42%
PdS_2_ QDs	0.027	850 509	1.47	11.11%
PdSe_2_ QDs	0.034	619 276	1.47	7.18%

**Fig. 7:**
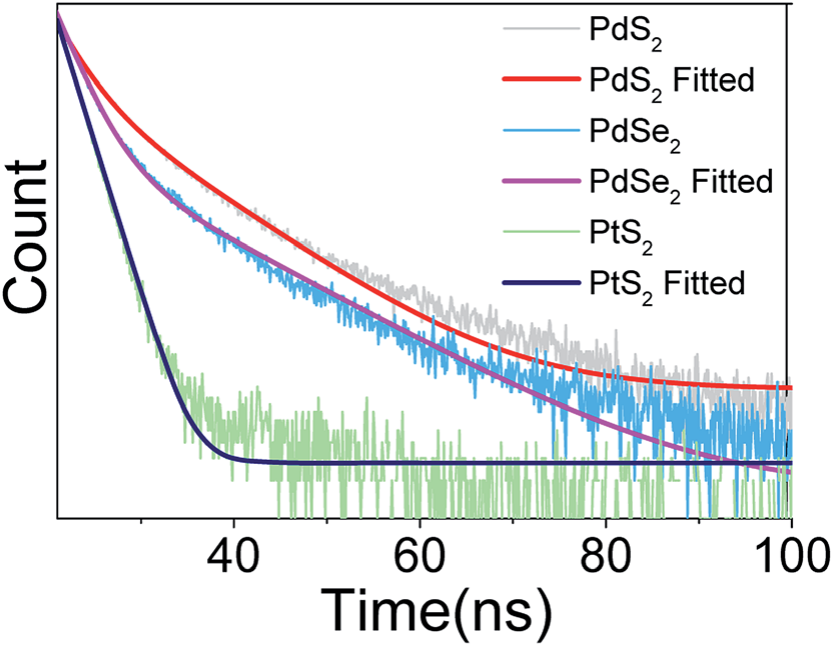
Decay profile of PdS_2_ QDs, PdSe_2_ QDs, and PtS_2_ QDs solutions.

In the next step, the time-resolved PL decay measurement is performed at room temperature as depicted in Fig. 7, while PdS_2_, PdSe_2_, and PtS_2_ QDs samples are excited by 377, 397.5, and 346 nm wavelengths, respectively. The decay lifetime curves of QDs samples are monitored and fitted by dual-exponential function. The function presented in eqn (2) explains that the decay curve is dominated by two de-excitation processes in the surface states and the core states, corresponding to a long-lived component *τ*_1_ and a short-lived component *τ*_2_.^53,54^ The average lifetime (*τ*_ave_) is estimated by substituting parameters of eqn (2) into eqn (3). The fitted values as listed in Table 2 suggest that *τ*_1_ dominates the decay lifetime for all QDs samples, indicating that their core states play essential role in the PL decay kinetics. The Pd based QDs may exhibit prolonged decay lifetimes than the Pt based group-10 TMDs QDs according to *τ*_ave_. The better stabilities of PdS_2_ and PdSe_2_ may attribute to this longer decay lifetime.2
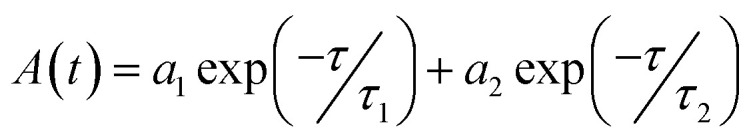
3
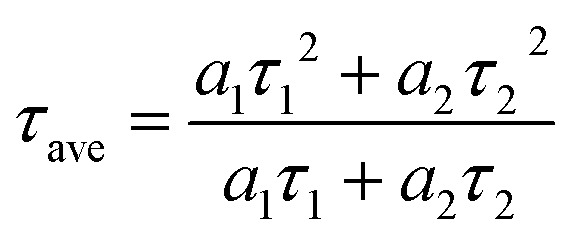


**Table 2:** Fitted decay lifetimes for PtS_2_ QDs, PdS_2_ QDs, and PdSe_2_ QDs solutions

	*a*_1_	*τ*_1_/ns	*a*_2_	*τ*_2_/ns	*τ*_ave_/ns
PtS_2_ QDs	8.85 × 10^8^	0.15	9.32 × 10^8^	1.81	1.69
PdS_2_ QDs	25 672	9.06	1.77 × 10^7^	2.63	2.67
PdSe_2_ QDs	6.33 × 10^7^	3.64	6024	10.93	2.37

## 4. Conclusions

In this study, 2 typical group-10 TMDs materials QDs were fabricated *via* solvent exfoliation and investigated by comparing with a different group-10 TMDs PtS_2_ QDs. The as-prepared PdS_2_ and PdSe_2_ QDs exhibit average diameters of 4.99 and 4.17 nm, while their average thicknesses were determined as 2.61 and 1.99 nm. Very good air stabilities were observed for PdS_2_ and PdSe_2_. The optical characteristics for those QDs solutions were monitored, exhibiting excitation wavelength-dependent behavior and large stokes shifts. The PL emission wavelengths might be strongly related to the size of QDs due to quantum size effect. This study is basically a short demonstration of simple synthesis process using solvent exfoliation and several optical performance parameters for two different group-10 TMDs materials of PdS_2_ and PdSe_2_ QDs. Most importantly, to our knowledge, so far this is the first demonstration of PL behaviors for PdS_2_ and PdSe_2_ QDs. We believe that this study with some excellent optical properties of these noble 2D materials should be beneficial for applications related to real time imaging, single molecule detection, light-emitting diode, and bio-sensing in addressing some analytical and biological issues which are currently being encountered in the fields.

## Conflicts of interest

There are no conflicts to declare.

## Supplementary Material

RA-009-C9RA07445G-s001
