# Supplementary material for: Photoluminescence of PdS2 and PdSe2 quantum dots
Source: RSC Adv. 2019 Nov 21;9(65):38077–84. doi: 10.1039/c9ra07445g (PMC9075810; doi:10.1039/c9ra07445g)
Supplement: RA-009-C9RA07445G-s001 [file RA-009-C9RA07445G-s001.pdf]

Electronic Supplementary Material (ESI) for RSC Advances.
This journal is © The Royal Society of Chemistry 2019

ELECTRONIC SUPPLEMENTARY INFORMATION (ESI)

Photoluminescence of PdS_2 and $PdSe_2$ Quantum Dots

Xinyu Wang[a,b], Wayesh Qarony[a,b], Ping Kwong Cheng[a,b], Mohammad Ismail[a,b] and Yuen Hong Tsang[*a,b]

[a.] The Hong Kong Polytechnic University Shenzhen Research Institute, Shenzhen, Guangdong, China.

[b.] Department of Applied Physics and Materials Research Centre, The Hong Kong Polytechnic University, Hung Hom, Kowloon, Hong Kong, China.

[*]Corresponding author, Email: yuen.tsang@polyu.edu.hk.

PtS_2 quantum dots (QDs) characterizations

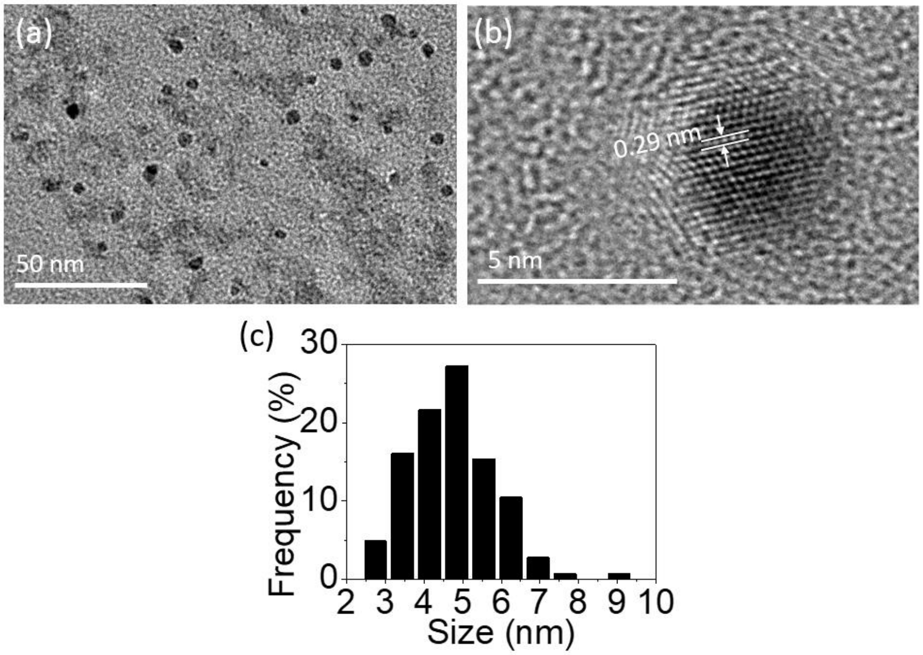

Fig. S1 (a) TEM image; (b) high resolution TEM image; and size distributions based on multiple images of PtS_2 QD.

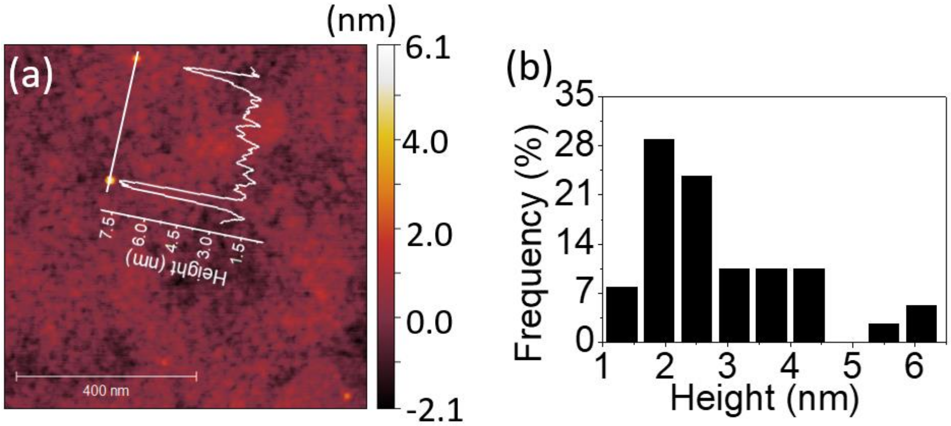

Fig. S2 (a) Line profile and corresponding AFM image; and (b) height distribution of PtS_2 QDs.

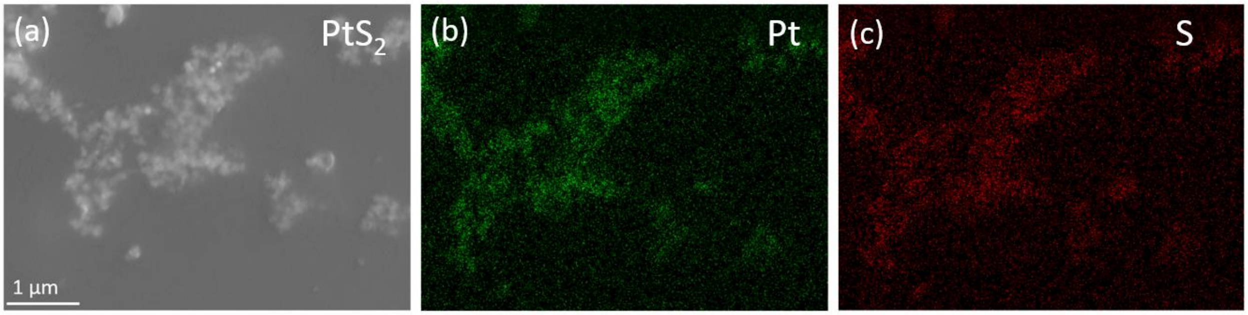

Fig. S3 (a) SEM image of PtS_2 QDs solution drop-casting on silicon substrate; (b-c) EDX elemental maps of Pt and S elements in (a).

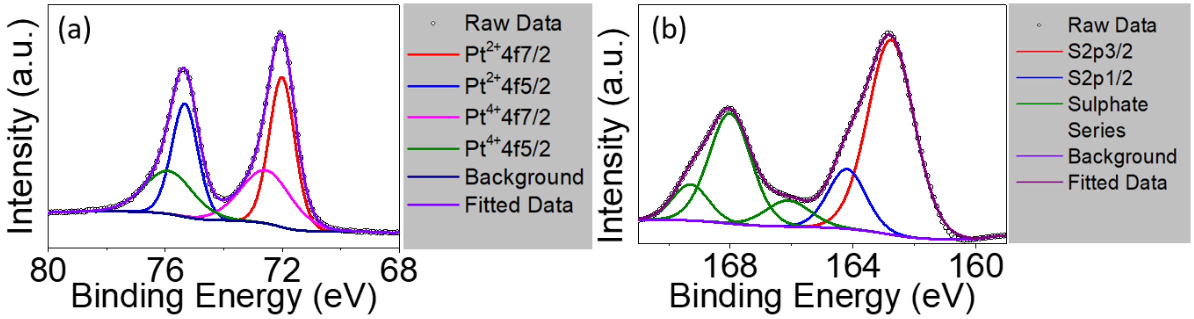

Fig. S4 High resolution X-ray photoelectron spectra of PtS_2 for (a) Pt4f region and (b) S2p region.

PtS_2 QDs optical performances and analyzations

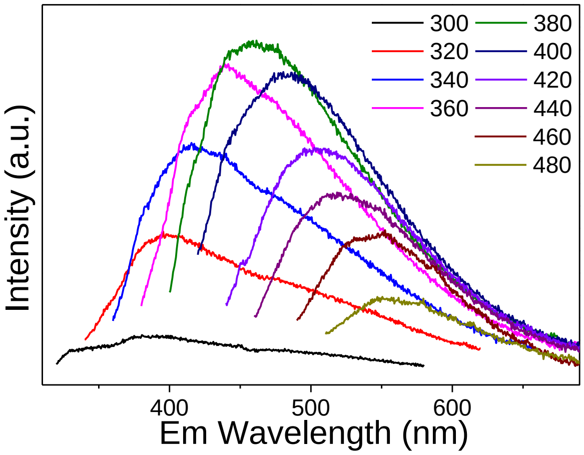

Fig. S5 Emission (Em) spectra of PtS_2 QDs solution.

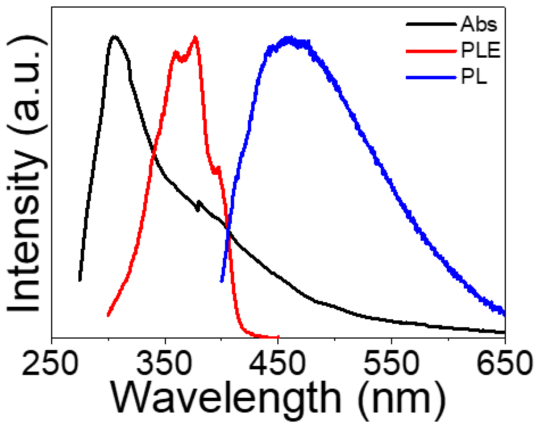

Fig. S6 Normalized absorption spectra (black curve), and photoluminescence excitation (PLE) spectra (red curve) for PtS_2 QDs solution at 468.5 emission wavelengths, which is observed at the peaks of the corresponding emission spectra (blue curve).
